# Gentle Handling Attenuates Innate Defensive Responses to Visual Threats

**DOI:** 10.3389/fnbeh.2018.00239

**Published:** 2018-10-18

**Authors:** Xuemei Liu, Chen Chen, Yuanming Liu, Zhijie Wang, Kang Huang, Feng Wang, Liping Wang

**Affiliations:** ^1^Shenzhen Key Lab of Neuropsychiatric Modulation and Collaborative Innovation Center for Brain Science, CAS Center for Excellence in Brain Science and Intelligence Technology, The Brain Cognition and Brain Disease Institute, Shenzhen Institutes of Advanced Technology, Chinese Academy of Sciences, Shenzhen, China; ^2^University of Chinese Academy of Sciences, Beijing, China

**Keywords:** gentle handling, innate fear, sensory input, superior colliculus, overhead looming stimulus

## Abstract

Innate defensive responses to threats are essential for animal survival. The complexity and variability of innate defensive behaviors can be due to individual experiences, environmental factors, and internal states. However, it is not completely understood if the gentle handling involved in sensory processing affects innate defensive responses to visual threats. Here, we report attenuation of innate defensive responses after gentle handling accompanied by de-excitation of the intermediate layer (IL) and deep layer (DL) of the superior colliculus (SC) but not of the superficial layer (SL). Our theoretical analysis of the c-Fos network revealed an increased correlation in module 1, which maybe generally functionally associated with fear emotional, a decreased correlation in module 2, which maybe generally functionally associated with sensory processing. The IL of the SC appeared to have the highest correlation with the two modules. We verified the dynamic activities of the IL of SC in response to overhead looming stimulus using fiber photometry. Retrograde labeling of 18 regions of interest (ROIs) showed that the IL received significant inputs from the cortical areas, thalamus, hypothalamus, and brainstem. These data suggest the sensory processing involved in the modulatory roles of the SC in innate fear processing.

## Introduction

Innate defensive responses to threats are essential for animal survival (LeDoux, 2012; Anderson and Adolphs, 2014; Janak and Tye, 2015; Tovote et al., 2015). Although defensive behaviors are stereotyped, their complexity and variability can be due to individual experiences (Morgan, 1896), environmental factors, and internal states (LeDoux, 2012; Anderson and Adolphs, 2014; Anderson, 2016). However, whether sensory input regulate the innate fear behavioral output is not well defined.

Overhead looming stimuli that mimic aerial predators have been shown to trigger stereotyped defensive responses across species (Yilmaz and Meister, 2013; Muijres et al., 2014; Temizer et al., 2015). Recently, looming-evoked defensive behavior and its underlying neural circuitry via the superior colliculus (SC) have received increasing attention (Yilmaz and Meister, 2013; Shang et al., 2015; Wei et al., 2015; De Franceschi et al., 2016; Huang et al., 2017; Vale et al., 2017; Evans et al., 2018; Li et al., 2018; Salay et al., 2018). SC is a laminated retinal recipient structure, of which its superficial layer (SL) receives direct retina input and primarily responds to visual stimuli. The intermediate layer (IL) and deep layer (DL) of the SC contain neurons that respond to multimodal (somatosensory, auditory, visual) sensory inputs (Wurtz and Goldberg, 1971; Stein, 1984).

Laboratory housing, husbandry, and handling methods have been shown to affect the internal state of the animals (Grandin, 1997; Poole, 1997; Deacon, 2006; Hurst and West, 2010; Clarkson et al., 2018). Unpleasant somatosensations such as tail handling and rough handling reduce the response to reward and increase stress levels (Grandin, 1997; Clarkson et al., 2018). Conversely, a gentle touch plays an essential role in the pleasant somatosensory processing conserved across species (Lumpkin et al., 2010; Abraira and Ginty, 2013). In mammals, a gentle touch is the expression of preference between mates, parenting, and affiliation (Barnett, 2005; Ebisch et al., 2016). It has also been demonstrated that massage has positive effects on health in human, grooming reduces stress levels in primates, and tactile stimulation alleviates the effects of stress in fish (Field et al., 2005; Soares et al., 2011). Our previous study demonstrated that stress accelerates the defensive responses to looming in mice (Li et al., 2018). However, it remains unknown if the repeat pleasant somatosensation of gentle handling affects innate defensive behaviors to visual threats.

Here, we found that gentle handling attenuated innate defensive responses to overhead looming. In addition, the IL and DL (but not the SL) of the SC had significantly reduced activities in response to overhead looming after repeated gentle handling. Functional analysis of the c-Fos network revealed that the highest correlation was between the IL of the SC in two modules among the 16 ROIs. Moreover, we verified the dynamic activities of the IL of SC to overhead looming stimulus using fiber photometry. Retrograde labeling of 18 ROIs showed that the IL had significant inputs from the cortical areas, thalamus, hypothalamus, and brainstem, suggesting the potential modulatory roles of the SC in innate fear and sensory processing.

## Materials and Methods

### Animals

All husbandry and experimental procedures in this study were approved by the Animal Care and Use Committee at the Shenzhen Institute of Advanced Technology, Chinese Academy of Sciences (Shenzhen, China). Male (6 weeks of age) C57BL/6 mice obtained from Beijing Vital River Laboratory Animal Technology Co., Ltd. (Beijing, China) were kept in a quiet room (22–25°C) with a 12 h light-dark cycle, and were given food and water *ad libitum*.

### Viral Injection

Mice were anesthetized with intraperitoneal (i.p.) injection of pentobarbital sodium (0.5% w/v, 80 mg/kg) and placed in a stereotaxic frame (RWD Life Science, Shenzhen, China). Microinjection needles were inserted unilaterally directly above the IL of the SC, using the following coordinates from Bregma: AP, -3.85 mm, ML, ±0.8 mm, and DV, -1.85 mm. The cholera toxin subunit B (CTB) Alexa Fluor 594 Conjugate and adeno-associated virus (AAV) expressing EGFP driven by a neuron calcium/calmodulin-dependent kinase II (CaMKII) promoter (AAV9-CaMKIIα-GCaMP6-EGFP; BrainVTA Co., Ltd., China) were injected using a 10 μL microsyringe with a 33 gauge metal needle (Hamilton; Sigma, United States) connected to the UltraMicroPump 3 microsyringe pump injector (World Precision Instruments, United States) and its controller (Micro4; World Precision Instruments) at a rate of 100 nL/min. After injection, the needle was left in place for an additional 10 min to allow diffusion of the virus particles away from the injection site, and then it was slowly withdrawn.

### Histology, Immunohistochemistry, and Microscopy

To quantify the expression of c-Fos positive neurons in the whole brain induced by overhead looming stimuli after consecutive 7 days of gentle handling or no-handled, the two group of mice were sacrificed 1.5 h after presentation of overhead looming stimuli on the 8th day. Mice were perfused by overdosing with chloral hydrate (10% w/v, 300 mg/kg, i.p.) and transcranially perfused with cold 1 M PBS followed by ice-cold 4% paraformaldehyde (PFA; Sigma) in 1 M PBS. Brains were removed and submerged in 4% PFA at 4°C overnight to post-fix, and then transferred to 30% sucrose to equilibrate. The coronal brains sections (40 μm) were obtained with a cryostat microtome (CM1950; Leica, Germany). Freely floating sections were washed with PBS, and blocked for 1 h at room temperature in blocking solution containing 0.3% Triton X-100 and 10% normal goat serum (NGS). Then the sections were incubated overnight with rabbit monoclonal anti-c-Fos (1:300, #2250; Cell Signaling Technology, United States) diluted in PBS with 3% NGS and 0.1% TritonX-100. The sections were incubated for 1 h at room temperature with Alexa Fluor 488 goat anti-rabbit secondary antibody (1:200; Jackson Laboratory, United States). Finally, the sections were mounted, coverslipped with DAPI (1:50,000, #62248; Thermo Fisher Scientific, United States), and photographed using the Olympus VS120 virtual microscopy slide scanning system (Olympus; Japan). The images were acquired using identical gain and offset settings, and analyzed with ImageJ, Image Pro Plus, and Adobe Photoshop software. ROIs were traced with reference to the “The mouse brain in stereotaxic coordinates, George Paxinos and Keith B. J. Franklin,” and c-Fos immunoreactivity was quantified using Image Pro Plus that was checked by comparing to manual counts by a trained double-blind observer.

### Behavior Assay

#### Gentle Handling

After obtained from the laboratory animal center, the mice have 1 week to habituation to the new animal facility and cage homes. Gentle handling was performed in the arena.

Briefly, mice were placed individually on a piece of medical absorbent cotton for 3 min handling once per day for 7 consecutive days. Handling sessions were performed by gently and thoroughly touching the mouse body through the cotton. New cages with the previous bedding were used to house the mice that had been handled. Handling sessions were performed in the same room by the same experimenter. The mice in the non-handled group received the same approach without the handling treatment, including habituation to the looming box on the 7th day and receive three times upper visual looming stimuli on the 8th day.

#### Looming Stimulation Test

The behavioral arena was a 40 cm × 40 cm × 30 cm closed plexiglass box with a shelter nest in the corner and an LCD monitor on the ceiling to present the looming stimulus (LS). The LS was a black disk that expanded from 2 to 20 degrees in diameter; it was presented 15 times in 5.5 s. The stimulation was manually triggered by the experimenter when the mice were in the farthest area away from the shelter. The handling group of the mice have experienced the recurrent 7 days gentle handling. On the 7th day, the mice in gentle handled group and non-handled group have 10 min to habituation to the looming box. Then on the 8th test day, after 3 min pre-test session, all the mice have subjected three times of the upper visual black looming stimulation. And the duration between each of the stimulation is no less than 3 min.

### Fiber Photometry

A fiber photometry system (ThinkerTech, Nanjing) was used to record the calcium signals of CaMKIIα-positive neurons in the IL of the SC in response to upper visual field looming stimulation. Two to 3 weeks following AAV9-CaMKIIα-GCaMP6-EGFP injection into the IL, an optical fiber (220 μm O.D., 0.37 NA; Newdoon, China) was placed above the IL of the SC. One week after the surgery, mice took 10 min to habituation to looming box the day before the test day. In the test day, mice received three times upper visual field black looming stimulations and three times upper visual field white looming stimulations. The upper visual field white looming here is used as a control which does not evoke the flight behavior.

To record the fluorescence signals, a 480 nm excitation light from a LEDs (CREE XPE), reflected of a dichroic mirror with a 435–488 nm reflection band and a 502–730 nm transmission band (Edmunds Inc.) and coupled to a long optical fiber (220 μm O.D., 0.37 NA, 2 m long, Thorlabs, Inc.). The laser intensity at the fiber tip was 20–30 μW to minimize GCaMP bleaching. GCaMP fluorescence was filtered with a GFP bandpass (Filter 525/39; Thorlabs, Inc., United States), detected by the sensor of a CMOS camera (Thorlabs, Inc. DCC3240M). A Lab view program is developed to control the CMOS camera and acquire calcium signal in about 50 Hz. The behavior event signal is recorded by a DAQ card (NI, usb-6001) in 1000 Hz using the same program.

### Statistical Analysis

All data values are presented as the mean ± SEM. Mann–Whitney *U* test was used to analyze statistical differences using GraphPad Prism 7 software. Statistical significance was set at ^∗^*p* < 0.05, ^∗∗^*p* < 0.01, ^∗∗∗^*p* < 0.001, ^∗∗∗∗^*p* < 0.0001. Functional network analyses of the c-Fos dataset were computed with the Rubinov and Sporns (2010) Brain Connectivity Toolbox^1^ in MATLAB R2016 (The Mathworks Inc.) Graph theoretical analysis of c-Fos data between gentle handling and non-handling conditions was used to calculate the relationship among the 16 ROIs. Correlations among the 16 ROIs arranged by rank-order were conducted using Kendall’s Tau correlation coefficient. Community correlation network and participation coefficients were analyzed according to previous studies, and a threshold value of 0.35 was set (Newman, 2006; Power et al., 2013; Vetere et al., 2017; Rogers-Carter et al., 2018).

## Results

### Gentle Handling Attenuates Defensive Responses to Overhead LS

**FIGURE 1:**
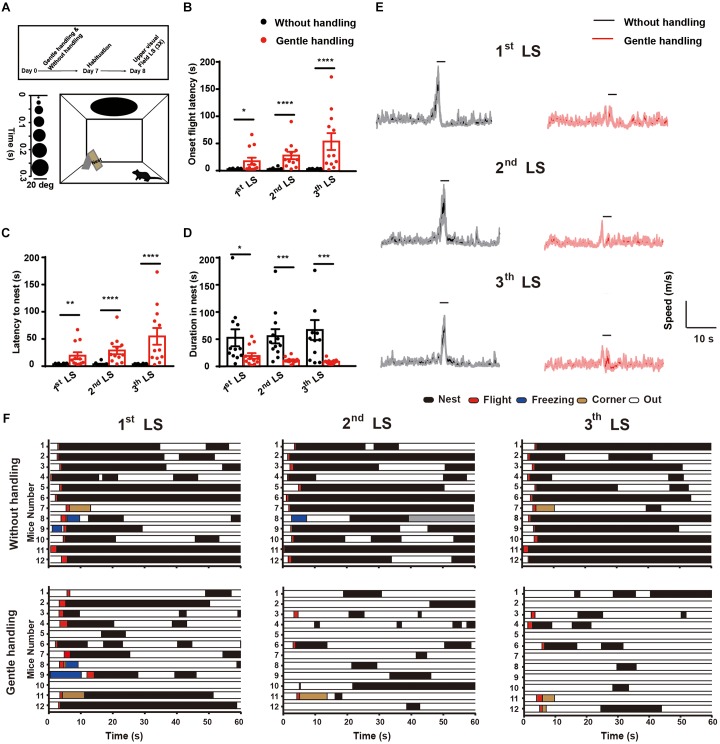
**(A)** Schematic diagram showing the behavioral arena and dark disk expansion from 2–20 degrees for 15 repeated cycles in 5.5 s. Timeline showing the design of the gentle handling method (days 1–7, 3 min/day). On the test day (day 8), LS was presented for 15 repeated cycles in 5.5 s with intervals of at least 3 min. After three trials with overhead LS, Mann–Whitney *U* tests showed that the gently handled group had longer latency to **(B)** onset flight behavior (*U* = 31, *p* = 0.0165; *U* = 5, *p* < 0.0001; *U* = 9, *p* < 0.0001 after 1st, 2nd, and 3rd LS, respectively) **(C)** latency of flight to nest (*U* = 21, *p* = 0.0022; *U* = 5, *p* < 0.0001; *U* = 6.5, *p* < 0.0001 after 1st, 2nd, and 3rd LS, respectively), and **(D)** decreased duration hiding in the nest (*U* = 32, *p* = 0.0205; *U* = 12, *p* = 0.0002; *U* = 17, *p* = 0.0008 after 1st, 2nd, and 3rd LS, respectively) compared with the non-handled group (*n* = 12 in each group; ^∗^*p* < 0.05, ^∗∗^*p* < 0.01, ^∗∗∗^*p* < 0.001, ^∗∗∗∗^*p* < 0.0001). Data are presented as the mean ± SEM. **(E)** The average locomotion speed of mice with gentle handling in the three trials of LS (horizontal black bar indicates duration of looming, *n* = 12 mice in each group, shaded area indicates SEM of the averaged data before and after 60 s). **(F)** Occurrence of flight, freezing, hiding in nest, and out of nest behaviors 1 min after 1st, 2nd, and 3rd LS presentation.

After 1 week habituation in the laboratory, mice underwent 7 consecutive days of gentle handling. To determine whether gently handling affects the innate defensive responses of mice, we used a behavioral assay with a rapidly expanding dark disk stimulus that mimicks an approaching predator (Yilmaz and Meister, 2013; Shang et al., 2015; Wei et al., 2015; De Franceschi et al., 2016; Huang et al., 2017; Vale et al., 2017; Salay et al., 2018). In response to repeated LS (three trials, intervals longer than 3 min), gently handled mice had an increased latency to onset flight behavior (**Figure 1B**), increased latency of flight to nest (**Figure 1C**), decreased duration hiding in nest (**Figure 1D**), and decreased locomotion speed (**Figure 1E**) compared with the non-handled group (*n* = 12 in each group, Mann–Whitney *U* test, ^∗^*p* < 0.05, ^∗∗^*p* < 0.01, ^∗∗∗^*p* < 0.001, ^∗∗∗∗^*p* < 0.0001). Taken together, these results demonstrate that long-term repeated handling attenuates innate defensive responses to visual threat stimuli. These data suggest that instinctive behavioral output is sensitive to tactile stimulation.

### Gentle Handling Reduces the Activities of SC Neurons in Response to Overhead Looming Stimulus

**FIGURE 2:**
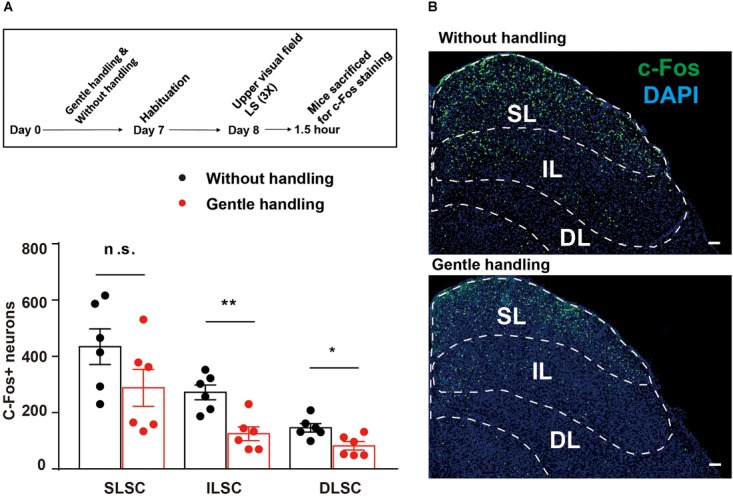
**(A)** The timeline and bar graph showing reduced c-Fos expression in the gently handled group compared with the non-handled group in response to LS. **(B)** Representative images showing c-Fos expression in the SC of gently handled or non-handled groups in response to LS (SL, superficial layer of SC; IL, intermediate layer of SC; DL, deep layer of SC); Scale bars = 100 μm. Mann–Whitney *U* test, ^∗∗^*P* < 0.01, *U* = 2; ^∗^*P* < 0.05, *U* = 4.

To test whether gentle handling affects SC responses to LS, we performed c-Fos labeling in the gently handled and non-handled groups after the behavioral test. The gently handled group had attenuated innate defensive behavior (**Figure 1**) accompanied by a decrease of c-Fos expression in the IL and DL of the SC, but not in the SL of the SC (**Figure 2**; *n* = 6 mice in each group, Mann–Whitney *U* test, *U* = 8, *p* = 0.132, *U* = 2, ^∗∗^*p* = 0.0087, *U* = 4, ^∗^*p* = 0.0260). These data indicate that gentle handling reduced the activities of the IL and DL of the SC in response to the black looming in the upper visual field.

### Analyses of the c-Fos Functional Network in Response to Overhead Looming Stimulus After Gentle Handling

**FIGURE 3:**
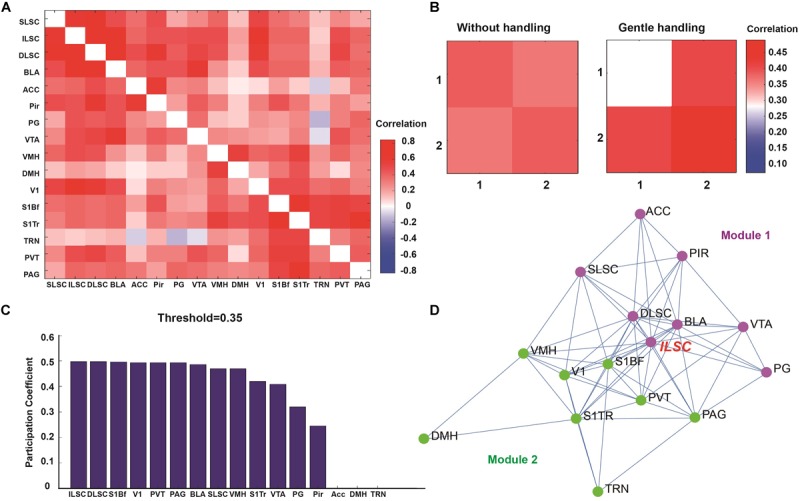
**(A)** Correlation matrix showing the functional correlation among ROIs, SLSC (superficial layer of superior colliculus), ILSC (intermediate layer of superior colliculus), DLSC (deep layer of superior colliculus), BLA (basolateral amygdala), ACC (anterior cingulate cortex), Pir (piriform cortex), PG (pontine gray), VTA (ventral tegmental area), VMH (ventromedial hypothalamic nucleus), DMH (dorsal hypothalamic area), V1 (primary visual cortex), S1BF (primary somatosensory cortex, barrel field), S1TR (primary somatosensory cortex, trunk region), TRN (tegmental reticular nucleus), PVT (paraventricular thalamic nucleus), PAG (periaqueductal gray matter). **(B)** Two modules were classified by graph theoretical analysis. **(C)** Participation coefficients were calculated to determine the degree of each node connected to multiple networks. **(D)** Functional network nodes were exhibited by network visualization (module 1 is in purple, module 2 is in green).

To determine the functional network connectivity, we performed c-Fos mapping in 16 ROIs in response to overhead LS between gently handled and non-handled mice. The 16 ROIs are selected by the expression of c-Fos which is comparatively higher than some other brain regions, also the brain regions what we choose, is most like generally functionally associated with the “fear” and “sensory” processing. Graph theoretical analysis was used to divide the 16 ROIs into two modules, in which the functional correlation of ROIs in each module was higher than that between modules (**Figure 3A**).

Our paradigm used in the current study is overhead life-threatening looming stimulation which mimic the predatory in the upper visual field. And most ROIs in module 1 (**Figure 3D**, purple dots) maybe generally functionally associated with “fear” related, such as different layer of superior colliculus, ventral tegmental area, basolateral amygdala, anterior cingulate cortex, pontine gray, piriform cortex. And the recurrent long-term (7 days continuously stimulation) gentle handling should transmit sensory input signal. And most ROIs in module 2 (**Figure 3D**, green dots) maybe generally maybe generally functionally associated with “sensory” related, such as primary somatosensory cortex, barrel field; primary somatosensory cortex, trunk region; primary visual cortex. After gentle handling, the correlation was decreased in module 1 and increased in module 2, and the correlation across modules 1 and 2 was increased (**Figure 3B**). The participation coefficients were calculated to determine the hubs in the functional correlation-based networks (Power et al., 2013; Vetere et al., 2017; Rogers-Carter et al., 2018). The nodes with the highest participation coefficients across two modules included brain areas such as the IL and DL of the SC, S1BF, V1, PVT, and PAG (**Figure 3C**). Functional network analysis indicated that compared to the SL and DL, the IL of the SC was more densely connected with other nodes related to innate fear and sensory perception.

### Dynamic Activation of the IL of SC in Response to Looming Overhead Stimulus

**FIGURE 4:**
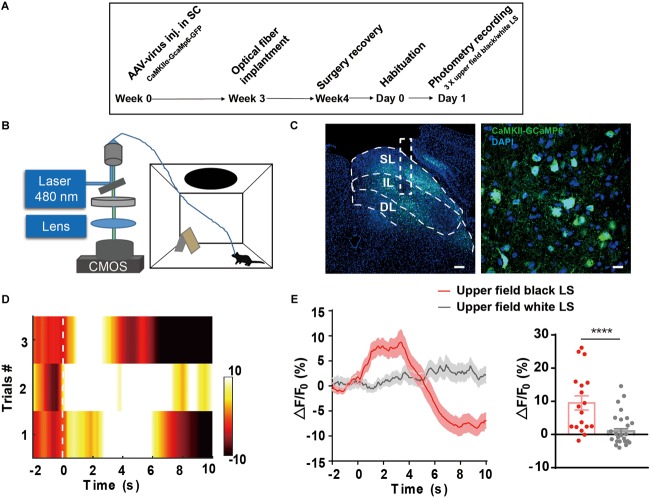
**(A,B)** The timeline and schematic of fiber photometry recording of LS-induced Ca^2+^ transient. **(C)** AAV9-CaMKIIα-GCaMP6-GFP expression in the IL of the SC; Scale bars, left = 100 μm; right = 10 μm. **(D)** Representative heatmap exhibits LS-induced Ca^2+^ transient across three trials. **(E)** Mean GCaMP signal response to black looming in the upper visual field and white looming (*n* = 6 mice in black looming, *n* = 5 mice in white looming, ^∗∗∗∗^*p* < 0.0001, Mann–Whitney *U* test, *U* = 117).

The SC, especially the SL of the SC, receives direct retinal input and mediates visual threat processing (Shang et al., 2015, 2018). To investigate whether the IL of the SC receiving somatosensory input reflects visual threat signals, we injected AAV9-CaMKIIα-GCaMP6s into the SC and optical fibers were implanted into the IL. Fiber photometry was used to record calcium transients in CaMKIIα-positive neurons in the IL in response to a black looming in the upper visual field (**Figure 4A**). A white looming in the upper visual field was used as a control, as it does not trigger flight-to-nest behavior (**Figures 4B,C**). The IL of SC CaMKIIα-positive neurons exhibited a significant increase in the activity after presentation of a black LS in the upper visual field (9.52 ± 2.13%, ΔF/F mean) compared with the white LS in the upper visual field (0.98 ± 0.74%, ΔF/F mean). Calcium signal onset to looming overhead stimulus rapidly increased (15% of peak signal as the onset time point) with a latency of 0.71 ± 0.18 s and peak signal of 19.08 ± 3%, ΔF/F (**Figures 4D,E**). Taken together, these data demonstrate the IL of SC CaMKIIα-positive neurons encode visual threat processing.

### Retrograde Labeling of Input to the IL of the SC

**FIGURE 5:**
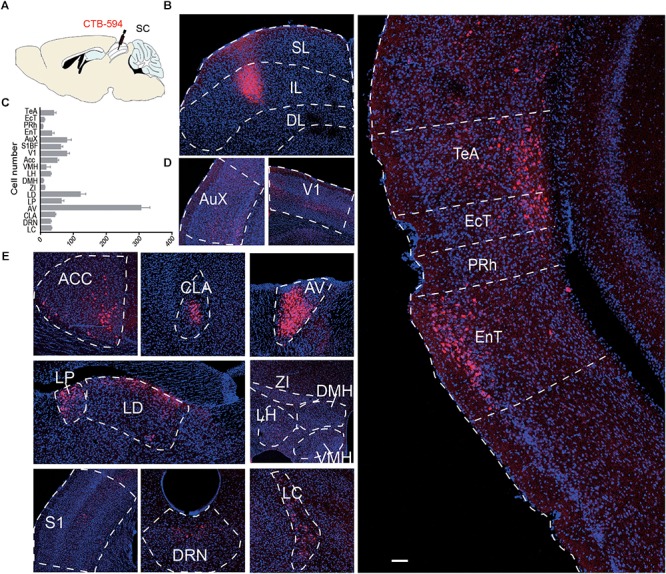
**(A)** Schematic diagram and **(B)** representative image of CTB expression in the SL and IL of the SC. **(C)** Bar graph showing the number of input neurons in each area (*n* = 4); **(D,E)** Representative area of input neurons. AuX, auditory cortex; V1, primary visual cortex; ACC, anterior cingulate cortex; CLA, claustrum; AV, Anteroventral thalamic nucleus; LP, lateral posterior thalamic nucleus; LD, laterodorsal thalamic nucleus; ZI, zona incerta; VMH, ventromedial hypothalamic nucleus; LH, lateral hypothalamic area; DMH, dorsal hypothalamic area; S1, primary somatosensory cortex; DRN, dorsal raphe nucleus; LC, locus coeruleus; TeA, temporal association cortex; EcT, ectorhinal cortex; PRh, perirhinal cortex; EnT, entorhinal cortex. Scale bars = 250 μm.

To visualize inputs to the IL, the CTB Alexa Fluor 594 Conjugate CTB was used for tracing. Two weeks after microinjection of CTB into the IL, the brain was sectioned to 40 μm, and every third section was processed for subsequent analysis. Across the entire brain, the most abundant labeling was found in the cortex (Aux, V1, S1, ACC, TeA, EcT, PRh, EnT), thalamus (LD, LP, AV), and hypothalamus (LH, DMH, VMH, ZI), brainstem (LC, DRN) (**Figure 5**). These data suggest that the IL of the SC receives broad cortical inputs from visual cortex, auditory cortex, and somatosensory cortex, as well as autonomic inputs from LC, LH, and thalamic nuclei (Saper, 2002).

## Discussion

The innate defensive reactions to threats are vital for survival. Although innate fear is instinctive and unconditional, the expression of innate fear can be affected by the circuits of motivation, arousal, emotional state, and reinforcement (LeDoux, 2012; Anderson and Adolphs, 2014). Tactile stimuli is a fundamental form of sensory perception conserved across species (Ratcliffe, 2012). Gentle handling has been shown to reduce stress, modify cognitive behavior, and improve the relationship between handlers and animals (Harlow and Suomi, 1970; Antoniazzi et al., 2017). Furthermore, gentle handling has been proven to decrease depression and reduce anxiety-like behavior in laboratory mice (Grandin, 1997; Madruga et al., 2006). Visually evoked defensive responses to overhead looming stimulus are initiated from the SC and lay downstream of the SC (Wei et al., 2015; Shang et al., 2018), but whether gentle handling affects innate visual threat processing is largely unknown.

In the current study, we demonstrated that gentle handling attenuates innate defensive reactions to visual threat stimuli, consistent with findings from previous studies (Grandin, 1997; Madruga et al., 2006). These studies provide a possibility that gentle handling might change the social interaction with experimenter and animal to improve the animal’s performance. Moreover, it’s possible that gentle handle induced the neurotransmitters such as oxytocin and dopamine release or lower corticotropin releasing hormone (CRH) to reduce emotionality, promote calm, and increase the ability to address visual threat situation. (Harlow and Suomi, 1970; Fenoglio, 2006; Ellingsen et al., 2014; Scheele et al., 2014; Peled-Avron et al., 2016).

Our c-Fos network data analysis revealed that after gentle handling, the correlation became weaker in module 1, but was stronger in module 2, and the functional connection across modules 1 and 2 became much stronger, as seen in **Figure 4B**. This suggests that gentle handling reduces activity in fear emotional-related regions but enhances the sensory connection response to visual threat. Functional network analysis demonstrated the IL and DL of the SC, S1BF, V1, PVT, and PAG, these brain areas have the highest participation coefficients across two modules. IL and DL of SC are thought to be involving in defensive behavior and are also functionally as the multisensory integration center (Sahibzada et al., 1986; Mooney et al., 1992). And in our study, IL and DL of SC are vital hubs to process both the sensory and visual threatening signals. S1BF and V1 are a part of primary sensory cortex, previous studies have defined the S1BF encode the representation of a sensory stimulus shaped by associative fear learning and V1 inputs to SC increase the response magnitude to looming (Gdalyahu et al., 2012; Zhao et al., 2014). Our recurrent gentle handling supposed to enhance the cortical plasticity and change the connection between cortex and subcortex which could lead to a change of defensive responses respond to overhead looming. PVT is a part of thalamus, which is recognized as relay station to transfer the sensory information, has proved to contribute to threatening events and fear memory (Penzo et al., 2015). PAG has defined to orchestrates sensory and motor (Koutsikou et al., 2015) and initiation of escape to looming stimuli (Evans et al., 2018). Taken together, all these brain regions functionally as hubs in powerful way in sensory and fear related networks.

The SC is a laminate sensory-motor structure. The SL of SC is thought to process primarily visual signal, whereas the IL and DL of SC exert sensory responses and multimodal integration, contributing to saccadic eye movements and head movements (Wurtz and Goldberg, 1971; Stein, 1984). Our CTB retrograde tracing data indicated that the IL of the SC receives broad cortical and autonomic inputs. Another possible mechanism may be multimodal integration of the IL-SC, in which recurrent somatosensory stimuli enhance visual discrimination (Macaluso et al., 2000, 2002). Our c-Fos mapping data demonstrated that, gentle handling affects visual threat signals by preferentially influencing different layer of SC. That’s c-Fos expression in gently handled mice was significantly higher in the IL and DL of the SC but not in the SL of the SC compared with the non-handled group respond to overhead looming stimuli. In addition, our c-Fos functional network analysis confirmed that the IL has more intensive connections with the other regions than the DL and SL of the SC. However, the different functions of IL and DL have not been well defined. Thus, the neural mechanisms underlying interlaminar modulation of the SC needs further investigations (Mooney et al., 1992; Saito and Isa, 2005; Cohen and Castro-Alamancos, 2010; Ghitani et al., 2014).

The results of this study indicate that innate defensive responses are sensitive to external conditions, which are vital factors that influence animals’ behaviors by affecting the internal state of brain. Our data consistent with the previous studies suggest that the environmental condition (e.g., housing, husbandry, habituation, handling, transport) and standard of behavioral protocol are crucial for animal behavioral tests, especially the innate behavioral test (Grandin, 1997; Poole, 1997; Deacon, 2006; Baumans, 2009; Hurst and West, 2010; Clarkson et al., 2018).

Our current study revealed that gentle handling changes the activation of key brain regions that respond to danger signaling, indicating that these regions are vital for processing the interaction between sensory perception and fear emotion. The fact that recurrent sensory stimuli affect innate fear processing suggest that related neural circuits may be causally responsible for psychiatric diseases such as autism, of which the core characteristics are sensory and emotion deficits (Pernon et al., 2007; Cascio et al., 2012; Robertson and Baron-Cohen, 2017).

## Author Contributions

XL, LW, and FW designed the experiments. CC performed the behavioral tests. YL conducted the fiber photometry recording. CC, YL, and XL performed the histological studies. XL, CC, YL, ZW, and KH analyzed the data. XL, LW, and FW wrote the manuscript.

## Conflict of Interest Statement

The authors declare that the research was conducted in the absence of any commercial or financial relationships that could be construed as a potential conflict of interest.

## Footnotes

1 https://sites.google.com/site/bctnet/
